# Prevalence of malnutrition and associated factors among community-dwelling older persons in Sri Lanka: a cross-sectional study

**DOI:** 10.1186/s12877-018-0892-2

**Published:** 2018-08-30

**Authors:** H. D. W. T. Damayanthi, F. M. Moy, K. L. Abdullah, S. D. Dharmaratne

**Affiliations:** 1 Department of Nursing Sciences, Faculty of Medicine, University of Malaya, Kuala Lumpur, Malaysia; 2 Department of Nursing, Faculty of Allied Health Sciences, University of Peradeniya, Peradeniya, Sri Lanka; 3 Julius Centre University of Malaya, Department of Social and Preventive Medicine, Faculty of Medicine, University of Malaya, Kuala Lumpur, Malaysia; 4 Department of Community Medicine, Faculty of Medicine, University of Peradeniya, Peradeniya, Sri Lanka; 5 Department of Family and Community Medicine, College of Medicine, University of Kentucky, Kentucky, USA; 6 Department of Health Metric Sciences, School of Medicine, University of Washington, Seattle, USA

**Keywords:** Elderly persons, Malnutrition, Mini nutritional assessment, Prevalence, Sri Lanka

## Abstract

**Background:**

Malnutrition in older persons is a public health concern. This study aimed to estimate the prevalence of malnutrition and its associated factors among community-dwelling older persons in Sri Lanka.

**Methods:**

A cross-sectional study was conducted in the Kandy district, Sri Lanka. The nutritional status of older persons was assessed using the Mini Nutritional Assessment –Short Form (MNA­SF). A standardised questionnaire was used to record factors associated with malnutrition: demographic characteristics, financial characteristics, food and appetite, lifestyle, psychological characteristics, physical characteristics, disease and care, oral health, and social factors. Complex sample multinomial logistic regression analysis was performed.

**Results:**

Among the 999 participants included in the study, 748 (69.3%) were females and 251 (25.1%) were males. The mean age was 70.80 years (95% CI: 70.13, 71.47). The prevalence of malnutrition, risk of malnutrition and well-nutrition was 12.5%, 52.4% and 35.1% respectively. In the multivariate model, hypertension (adjusted OR = 1.71; 95% CI: 1.02, 2.89), alcohol consumption (aOR = 4.06; 95% CI: 1.17, 14.07), and increased age (aOR = 1.06; 95% CI: 1.01, 1.11) were positively associated with malnutrition. An increased number of people living with the older person (aOR: 0.91; 95% CI: 0.85, 0.97) was a protective factor among those at risk for malnutrition.

**Conclusion:**

Both the prevalence of malnutrition and risk of malnutrition were commonly observed among community-dwelling older persons in Sri Lanka. The associated factors identified in this study might help public health professionals to implement necessary interventions that improve the nutritional status of this population.

## Background

Sri Lanka is experiencing rapid growth of its older population due to the improvement of health care facilities. In the South Asian region, Sri Lanka has the fastest-growing ageing population [1]. By year 2041, one out of every four persons in Sri Lanka is expected to be an older person aged 60 years and older [2, 3]. Changes in physiological, pathological, social, and psychological conditions can be observed among older persons as they go through the process of aging [4]. Malnutrition among older persons is a key determinant of their mortality, health care and quality of life [5–8].

Malnutrition is often defined as ‘a faulty or inadequate nutrition status’ [9]. Malnutrition causes adverse effects on health as well as the quality of life of older persons. Further, it presents high costs to health care systems [10, 11]. Hence, malnutrition has become an important component of geriatric care that warrants monitoring.

The prevalence of malnutrition among older persons varies according to the setting in which they reside. In Sri Lanka, the prevalence of malnutrition among older persons who resided in nursing homes was 30% [12]. It ranged from 21 to 67% in the hospital setting [13]. However, there is a lack of literature on malnutrition among community-dwelling older persons in Sri Lanka.

Malnutrition among older persons is a multifactorial condition. A recent systematic review identified several domains of malnutrition: demographic characteristics, financial characteristics, food and appetite, lifestyle, psychological characteristics, physical functioning, disease and care, oral health, and social factors [14]. Previous studies have identified additional potential risk factors of malnutrition related to the above domains: advanced age, female and single/widowed/divorced [15, 16], low education level [17], poverty [17, 18], loss of appetite, food allergies and eating/swallowing/tasting difficulties, [12, 19, 20], cigarette smoking and alcohol consumption [21, 22], betel nut chewing [23], lack of interest, low mood, frequent negative thoughts, loneliness, depression, dementia and low cognition [17, 24], physical inactivity [12], diseases [11], tooth loss and not wearing dentures [25] and social factors such as living alone [26].

As most studies were conducted in high-income countries, where lifestyles and foods differ from that in Sri Lanka [5, 6, 17], research studies need to be conducted in Sri Lanka to reflect the nutrition status of the population. In addition, the magnitude of malnutrition among older persons in community settings in Sri Lanka has been under-reported thus far. Factors associated with malnutrition among community-dwelling older persons in Sri Lanka need to be identified in order to determine suitable interventions that can improve their nutritional status [12]. Therefore, this study was conducted to determine the prevalence and associated factors of malnutrition among community-dwelling older persons in Sri Lanka.

## Methods

### Study design and sample

This cross-sectional study was conducted to determine the prevalence and associated factors of malnutrition among community-dwelling older persons in Sri Lanka. A total of 1267 respondents were recruited and 1015 of them completed the questionnaires (response rate of 80.1%). Sixteen cases were subsequently excluded because of missing data. The final sample used for analysis was 999.

This study was conducted in Kandy district, Sri Lanka from July 2015 to May 2016. The Open Source Epidemiologic Statistics for Public Health software (version 3.01) was used to calculate the sample size. Sample size was calculated using the odds ratios of selected factors associated with malnutrition among older persons in previous studies and the largest sample size was taken for the present study. Nazemi and colleagues (2015) reported that older persons with diabetes mellitus were twice more likely [(OR): 1.67; 95% confidence interval (CI): 1.1–2.4] to have malnutrition compared to older persons without diabetes. The estimated sample size was 1056. Considering the 20% non-response rate, 1267 older persons were needed for the sample size of the study. Participants were included in the study if they were aged 60 years and older, stayed in the community and consented to participate in the study. Exclusion criteria included being physically frail and/or mentally unable to answer the questionnaire. Participants were selected using multistage sampling from twenty-six Grama Niladari (GN) divisions, among seven randomly-selected Divisional Secretariats in Kandy district. At the GN level, a list of older persons was obtained from the 2012 electoral registers utilizing the simple random sample approach.

### Data collection and procedures

The selected participants completed the questionnaires at a community centre. All participants were informed about the aim and content of the study and each participant signed an informed consent form prior to data collection. Anthropometric measurements (height and weight) were recorded using standardized procedures.

The nutritional status was assessed using the Sinhala version of the Mini Nutritional Assessment – short form (MNA-SF), which is an instrument specifically designed for elderly people; it has been validated in many settings including hospitals, nursing homes and communities [27, 28]. The MNA-SF is simple, non-invasive, inexpensive, and easy to use. The questionnaire consists of six items: food intake, weight loss, mobility, psychological stress or acute disease, neuropsychological problems and body mass index (BMI) [27]. The following cut-off values were used: 0 to 7 for malnourished, 8 to 11 for risk of malnutrition, and 12 to 14 for well-nutrition [29].

Body mass index (BMI) was calculated to assess the nutritional status of participants. BMI is calculated as weight in kilograms (kg) divided by height in square meters (m^2^). The World Health Organization classifies BMI < 18.5 as underweight, BMI 18.5–24.9 as normal weight, BMI ≥ 25 as overweight [30]. Reliable measurements of height of older persons, however, can be difficult due to vertebral compression, loss of muscle tone and postural changes. Hence, BMI was considered only for the assessment of nutritional status [31].

Interviewer-administered questionnaires were used to collect data on all explanatory variables, including demographic characteristics, financial characteristics, food and appetite, lifestyle, psychological characteristics, physical functioning, disease and care, oral health, and social factors. Demographic factors were age, sex, marital status and level of education. Selected older persons were categorized into three groups by age: the young old (60 to 69 years), the middle old (70 to 79 years), and the very old (80 years and older) [32]. With respect to marital status, participants were categorised as married, single, or divorced/widowed. Based on level of education, participants were also classified into three groups: no formal education, primary education and secondary/tertiary education.

Participants’ financial status was determined by whether their last monthly income was below or above the poverty level, which was Rs. 3999 (Sri Lankan rupees). Living arrangements and the number of people living with the older person were considered as social factors. Participants were divided into three groups based on social factors: alone, living with children/spouse or living with relatives/friends.

Loss of appetite, eating/swallowing difficulties, food allergies and vegetarian diet were included under food and appetite. Lifestyle factors included alcohol consumption, cigarette smoking and betel chewing. Disease and care included suffering from chronic illnesses, usage of medicines, and chronic conditions such as diabetes mellitus, hypertension and hypercholesterolemia. Tooth loss and use of dentures were considered oral factors. For these four explanatory variables – food and appetite, lifestyle, disease and care, and oral factors, participants were classified based on self-reported responses of ‘yes’ or ‘no’.

Physical characteristics included physical activity, which was measured using the Sinhala version of the International Physical Activity Questionnaire (IPAQ) short form validated for Sri Lankans [33]. All participants were asked to answer the IPAQ, which consisted of four parts: the frequency and time spent on vigorous intensity activity, moderate intensity activity, walking for at least 10 min at one time, and sitting and/or lying down (excluding sleeping) [34]. Physical activity value was obtained in Metabolic Equivalent of Task (MET) minutes per week; values were obtained by multiplying the value of energy expenditure for the given physical activity in MET by the weekly frequency (days per week) and the time (minutes per day). MET values for vigorous physical activity, moderate physical activity and walking were 8.0, 4.0 and 3.3 respectively. For the purpose of the current study, participants were grouped into three categories based on physical activity; ‘inactive/low’, ‘moderately active’ and ‘highly active’. Participants with scores of < 600 MET minutes per week were considered low in physical activity; those with scores of 600–2999 MET minutes per week were moderately active; and those with scores of ≥3000 MET minutes per week were highly active. The validly and reliability of this questionnaire for elderly people also has been ensured [35].

The depression level of the participants was considered as a psychological factor. It was measured using the Sinhala version of the geriatric depression scale (GDS) [36]. The GDS-short form contains 15 items with a ‘yes’ or ‘no’ answer format. It has been tested for psychometric properties [37, 38]. Participants were considered depressed if they had scores of more than 8 on the GDS [39].

### Statistical analysis

Data analysis was performed using the IBM Statistical Program for Social Sciences (SPSS) version 21. This sampling utilized multistage sampling of Divisional Secretariats (DS), Grama Niladari (GN) Divisions and households. Households within clusters are more similar than households randomly sampled from the population as a whole [40]. Complex sample analysis with weighting was carried out. The weights were determined by the proportion of the respective stratum in the population divided by the proportion of that stratum in the sample (the inverse of the probability of selection). Weights were applied to correct for unequal selection probabilities of multistage sampling. We computed the weights for each person based on DS, GN and household levels.

Initially, descriptive analyses was conducted to describe socio-demographic characteristics. The mean and 95% confidence interval (CI) were calculated for continuous variables. Unweighted counts and weighted percentages for categorical variables were reported. Then the independent variables were compared between nutritional categories (malnourished, at risk of malnutrition and well-nourished) using one way analysis of variance for continuous variables and Chi-square tests for categorical study variables. Then, complex samples multinomial logistic regression analyses, in the form of univariate and multivariate, were used to examine the associations between malnutrition and risk of malnutrition and study variables. Well-nutrition was used as the reference category. Variables entered into the multivariate model were those found to have significant associations (*p* < .05) with malnutrition and risk of malnutrition in the univariate analysis, while those with *p* > .25 in the univariate analysis were excluded from the multivariate model [41]. The multivariate analysis was adjusted for gender and all other variables in the model. Crude odds ratios (OR) and adjusted odds ratios (aOR) with 95% confidence intervals (CI) were reported in univariate and multivariate analysis respectively. Significant level was pre-set at 0.05.

## Results

The participants’ mean age was 70.80 years (CI: 70.13, 71.47). Almost half of the participants (48.1%) were young old, 42.3% were middle old and 9.5% were very old. Two-thirds (71.9%) were females. The majority were Sinhalese (94.0%). About half of them (55.6%) had completed secondary or tertiary education, while 10.2% had no formal education. The majority (73.9%) of the participants earned an income below the poverty line and lived with family members (88.8%) (Table 1).

**Table 1:** Nutritional status of older persons according to demographic, social, financial, food and appetite, lifestyle, psychological, physical, disease & care, oral characteristics and BMI categories (*N* = 999)

Variable	Total *n* = 999 (n, weighted %)	Malnutrition, *n* = 151 (n, weighted %)	At risk of malnutrition, *n* = 485 (n, weighted %)	Well nutrition, *n* = 363 (n, weighted %)	*p* value
Demographic characteristics					
Age categories					0.104
Young old	542(48.1)	52(8.0)	267(50.5)	223(41.4)	
Middle old	375(42.3)	81(15.4)	178(53.5)	116(31.2)	
Old old	81(9.5)	18(12.5)	39(55.1)	24(27.9)	
Sex					0.256
Male	251(28.1)	42(12.7)	128(58.0)	81(29.3)	
Female	748(71.9)	109(12.3)	357(50.0)	282(37.7)	
Marital status					0.095
Married	840(79.4)	135 (13.8)	407(50.6)	298(35.7)	
Single	15(2.2)	0	10(90.7)	5(9.3)	
Divorced/widowed	144(18.4)	16(9.8)	68(53.7)	60(36.5)	
Level of education					0.435
No formal	76(10.2)	13(13.9)	44(60.8)	19(25.3)	
Primary	270(34.2)	40(12.3)	141(54.6)	89(33.0)	
Secondary/tertiary	653(55.6)	98(12.2)	300(48.2)	255(39.6)	
Financial characteristic					
Income					0.276
Below poverty line	678(73.9)	104(13.1)	321(50.6)	253(36.3)	
Above poverty line	321(26.1)	47(9.8)	164(59.4)	110(30.7)	
Social characteristics					
Living arrangements					0.256
Alone	89(10.4)	17(11.8)	47(53.3)	25(35.0)	
With family members	900(88.8)	133(12.6)	434(52.2)	333(35.1)	
With friends/relatives	10(0.8)	1(2.3)	4(61.1)	5(36.6)	
Food & appetite characteristics					
Loss of appetite					0.659
Yes	140(11.0)	26(11.2)	70(58.0)	44(30.7)	
No	859(89.0)	125(12.6)	415(51.7)	319(35.7)	
Eating/swallowing difficulties					0.935
Yes	125(10.7)	19(13.4)	63(49.9)	43(36.7)	
No	874(89.3)	132(12.3)	422(52.7)	32(34.9)	
Food allergies					0.521
Yes	73(6.7)	10(19.4)	31(48.9)	32(31.4)	
No	926(93.3)	141(11.9)	454(52.7)	331(35.4)	
Vegetarian					0.587
Yes	328(34.7)	52(13.3)	155(48.8)	121(37.8)	
No	671(65.3)	99(12.0)	330(54.3)	242(33.7)	
Lifestyle characteristics					
Alcohol consumption					0.016*
Yes	36(5.2)	9(31.4)	17(55.9)	10(12.7)	
No	963(94.8)	142(11.4)	468(52.2)	353(36.4)	
Cigarette smoking					0.069
Yes	28(3.5)	6(32.4)	15(52.8)	7(14.8)	
No	971(96.5)	145(11.7)	470(52.4)	356(35.9)	
Betel chewing					0.027*
Yes	269(46.3)	46(17.1)	131(49.7)	92(33.1)	
No	730(53.7)	105(8.4)	354(54.7)	271(36.8)	
Psychological characteristics					
Depression					0.378
Normal	413(36.7)	61(12.4)	203(51.8)	149(35.8)	
Depression	586(63.3)	90(12.5)	282(52.8)	214(34.7)	
Physical characteristics					
Physical activity					0.991
Low	164(15.4)	24(11.1)	80(52.9)	60(36.0)	
Moderate	729(68.4)	111(12.4)	349(52.6)	269(35.1)	
High	106(16.2)	16(14.2)	56(51.3)	34(34.5)	
Disease & care characteristics					
Chronic illness					0.325
Yes	503(55.0)	69(10.4)	248(56.1)	186(33.5)	
No	496(45.0)	82(14.1)	237(49.4)	177(36.5)	
Usage of medicines					0.110
Yes	568(60.1)	92(15.2)	275(50.1)	201(34.7)	
No	431(39.9)	59(8.4)	210(55.9)	162(35.7)	
Medical conditions					
DM					0.385
Yes	62(3.9)	8(7.6)	31(64.2)	23(28.2)	
No	937(96.1)	143(12.6)	454(51.9)	340(35.4)	
HPT					0.320
Yes	203(23.5)	28(8.5)	101(57.8)	74(33.7)	
No	796(76.5)	123(13.7)	384(50.8)	289(35.6)	
HCH					0.728
Yes	14(0.5)	3(15.3)	7(41.3)	4(43.4)	
No	985(99.5)	148(12.4)	478(52.5)	359(35.1)	
Oral characteristics					
Tooth loss					0.503
Yes	881(89.5)	137(13.1)	430(52.3)	314(34.7)	
No	118(10.5)	14(7.3)	55(53.7)	49(39.1)	
Use dentures					0.877
Yes	270(15.1)	46(12.0)	129(50.3)	95(37.7)	
No	729(84.9)	105(12.5)	356(52.8)	268(34.7)	
BMI categories					< 0.001*
Underweight	127(13.7)	78(44.7)	49(55.3)	0	
Normal	541(58.1)	71(10.9)	297(58.3)	173(30.8)	
Over weight	323(28.3)	2(0.4)	135(37.9)	186(61.7)	
Age, mean(CI)	70.80(70.13,71.47)	72.3(70.6,74.0)	70.90(69.9,71.7)	70.2(69.0,71.4)	0.140
Number of people living with (CI)	3.60(3.42,3.77)	3.4(3.1,3.8)	3.5(3.3,3.8)	3.8(3.5,4.1)	0.256

Data are presented as number & weighted percentage (%) or as mean & 95% confidence interval (CI)

Significance between groups was determined using chi-square test for categorical variables and one way ANOVA for continuous variables

*DM* diabetes mellitus, *HPT* hypertension, *HCH* hypercholesterolemia, *BMI* body mass index

Poverty line-Kandy district poverty line July 2015 Rs.3909

*p < 0.05 is significant

The mean MNA score was 10.45 (CI: 10.24, 10.67). Based on the MNA scores, 12.5% of the participants were malnourished, about half (52·4%) were at risk of malnutrition and one-third (35.1%) were well-nourished. Table 1 further shows the results of comparison of different nutritional status (malnourished, risk of malnutrition and well-nourished) across various factors. Alcohol consumption, betel chewing and BMI categories showed statistically significant differences with the nutritional status. With reference to BMI, 44.7% of the malnourished older persons were categorized as being underweight compared to 0% of the well-nourished.

In the univariate analyses, age, hypertension, cigarette smoking and alcohol consumption were significantly associated with malnutrition. The number of people living with the older person was statistically significant for risk of malnutrition (Table 2).

**Table 2:** Crude odds ratio (95% CI) of nutritional status according to associated factors in multinomial logistic regression

Variable	Risk of malnutrition, *n* = 485		Malnutrition, *n* = 151	*p* value
	OR (95% CI)	*p* value	OR (95% CI)	
Age	1.02(0.99, 1.05)	0.291	1.06(1.02,1.10)	0.008*
Gender	1.49(0.91,2.45)	0.110	1.32(0.65,2.68)	0.437
Male				
Level of education				
No formal	1.79(0.58,5.50)	0.311	1.98(0.90,4.34)	0.288
Primary	1.21(0.61,2.41)	0.581	1.36(0.85, 2.17)	0.297
Secondary/tertiary	Reference			
Income (Below poverty line)	1.29(0.69,2.41)	0.400	1.05(0.45,2.44)	0.909
Living arrangement				
Alone	1.17(0.48,2.83)	0.714	1.25(0.24,6.42)	0.782
With family members	5.56(0.63,53.0)	0.926	5.38(0.48,60.67)	0.273
With relatives/friends	Reference			
No of people living with	0.91(0.86,0.97)	0.006*	0.90(0.76,1.70)	0.216
Loss of appetite	1.28(0.80,2.05)	0.290	1.28(0.65,2.54)	0.456
Eating/swallowing difficulties	1.02(0.58,1.80)	0.931	1.06(0.51,2.24)	0.866
Food allergies	0.84(0.34,2.70)	0.685	1.14(0.45,2.88)	0.777
Vegetarian	0.88(0.45,1.70)	0.680	1.03(0.72,1.47)	0.843
Alcohol consumption	2.04(0.73,5.71)	0.167	4.60(2.35,8.98)	< 0.001*
Cigarette smoking	2.20(0.75–6.45)	0.141	4.70(2.21,9.99)	< 0.001*
Betel chewing	1.09(0.71,1.69)	0.669	1.65(0.93,2.92)	0.082
Depression				
Depression	1.05(0.68, 1.64)	0.823	1.04(0.54, 2.02)	0.906
Normal	Reference			
Physical activity				
Low	0.75(0.23,2.44)	0.631	0.99(0.45, 2.20)	0.983
Moderate	0.86(0.35,2.09)	0.737	1.01(0.54,1.90)	0.638
High	Reference			
Chronic illness	1.11(0.87,1.42)	0.385	0.81(0.40,1.64)	0.546
Usage of medicines	0.98(0.75,1.28)	0.901	1.47(0.87,2.49)	0.142
DM	0.87(0.36,2.07)	0.731	0.80(0.27,2.44)	0.686
HPT	1.02(0.81,1.29)	0.859	1.89(1.15,3.09)	0.014*
HCH	0.99(0.19,5.06)	0.992	0.64(0.16,2.58)	0.526
Tooth loss	1.17(0.47,2.87)	0.727	1.85(0.43,8.02)	0.390
Use dentures	0.96(0.65,--1.43)	0.840	1.16(0.64,2.13)	0.596

*DM* diabetes mellitus, *HPT* hypertension, *HCH* hypercholesterolemia, *BMI* body mass index, *OR* odds ratio, *CI* 95% confidence interval

Poverty line-Kandy district poverty line July 2015 Rs.3909

*p < 0.05 is significant

Reference category is well nutrition (*n* = 363); reference category for loss of appetite, eating/swallowing difficulties, food allergies, vegetarian, alcohol consumption, cigarette smoking, betel chewing, chronic illness, usage of medicine, DM, HPT, HCH, tooth loss, use dentures is ‘no’; reference for gender is female

Factors associated with malnutrition and at risk of malnutrition in the multinomial logistic regression analysis after adjusting for age, number of people living with, alcohol use, smoking, betel chewing, usage of medication and gender are shown in Table 3. Participants who consumed alcohol were found to be four times more likely (aOR = 4.06, 95% CI: 1.17, 14.07) to have malnutrition. In addition, older persons with hypertension had approximately 71% more chance of being malnourished (aOR = 1.71, 95% CI: 1.02, 2.89). Age remained independently associated with an increased risk of malnutrition with OR of 1.06 (95% CI: 1.01, 1.11). The number of people living with was a protective factor for being at risk of malnutrition (aOR: 0.91, 95% CI: 0.85, 0.97).

**Table 3:** Factors associated with risk of malnutrition and malnutrition after adjusted for confounders using multinomial logistic regression

Variable	Risk of malnutrition, *n* = 485 aOR (95% CI)	*p* value	Malnutrition, *n* = 151 aOR (95% CI)	*p* value
Age	1.02 (0.99,1.05)	0.268	1.06 (1.01,1.11)	0.020*
Gender (Male)	1.26(0.59,2.72)	0.533	0.95(0.33,2.68)	0.912
No of people living with	0.91 (0.85,0.97)	0.006*	0.89(0.74,1.09)	0.277
Usage of medication	0.97 (0.74,1.28)	0.827	1.47(0.82,2.66)	0.186
Alcohol consumption	1.63 (0.65,4.08)	0.281	4.06(1.17,14.07)	0.029*
Cigarette smoking	1.57 (0.75,3.29)	0.222	2.28(0.88,5.93)	0.087
Betel chewing	0.99 (0.66–1.50)	0.986	1.30(0.77,2.19)	0.304
HPT	0.99 (0.79,1.27)	0.994	1.71 (1.02,2.89)	0.044*

*HPT* hypertension; The reference category is well-nutrition (*n* = 363); Reference category for alcohol consumption, cigarette smoking, betel chewing, usage of medicine, HPT is ‘no’; Reference for Gender is female

Multinomial regression was used, with a removal probability of 0.25

Adjusted for gender and for all other variables in the model. * *p* < 0.05 is significant

Model Fit: R^2^ = 0.048 (Cox and Snell); 0.056 (Nagelkerke); 0.025 (McFadden)

## Discussion

This study was conducted among community-dwelling older persons in the Kandy district in Sri Lanka. About half of the participants were young old, while a small proportion consisted of very old participants. This pattern is similar to the age structure of the Sri Lankan population and represents the Sri Lankan older population [42]. The majority of the participants were of the Sinhala ethnic group and is comparable with the findings of other community based studies conducted in Sri Lanka [43, 44]. The education level of the participants also was consistent with the national levels reported, in that the larger proportion of the participants had secondary/tertiary education [45].

We found that the prevalence of malnutrition among community-dwelling older persons was 12.5%, with more than half of the population being at risk of malnutrition; this corroborates the findings of other studies in India which used MNA [46, 47]. Several studies conducted in South Africa, Portugal, and Korea using MNA as the measurement tool, reported prevalence of malnutrition among community-dwelling older persons as 10.4%, 5.6% and 10.5% respectively [5, 20, 48]. In comparison to these studies, the present study found a higher prevalence of malnutrition. A possible reason for this discrepancy may be the low economic conditions faced by Sri Lankans, which may be closely intertwined with the household food security of the older population. However, we feel that further detailed studies could propose more evidence based explanation for this finding.

In comparison to studies conducted among older persons in other care settings of Sri Lanka, the community-dwelling older persons of the current study were less likely to be malnourished [12, 13]. This may be due to the extended family support involved in their nutritional care. Older persons living in other settings, especially institutions, often lack family support; most institutions have set menus for three meals, which may be lacking in essential nutrients [12]. Another possible reason for the low prevalence of malnutrition in our study might be because the definitions and tools used to assess malnutrition are different from those used in other studies [12]. For instance, malnutrition among older persons who stayed in nursing homes was assessed using BMI [5].

In the multivariate analysis, age was associated with malnutrition. The physiological changes of aging directly affect the metabolism of nutrients. Also, physiological conditions of aging, such as sarcopenia and osteoporosis, might progressively limit the mobility of older individuals, further limiting their ability in shopping, preparing foods and even consuming foods [49]. This finding is similar to that reported in another study [4]. Our participants with hypertension were 70% more likely to be malnourished. The reason behind this may be the pathophysiological effects of diseases which lead to loss of appetite and decreased digestion, absorption and metabolism [50].

Alcohol consumption was also found to contribute to the prevalence of malnutrition. Alcohol and malnutrition may be linked to impaired liver function which directly affects protein metabolism [51]. Further, vitamin and mineral deficiencies associated with heavy alcohol consumption deteriorate the nutritional status of older persons [52]. However, there were contradictory findings on the association between alcohol consumption and malnutrition reported in other studies [22]. Tian et al. (2017) reported that moderate alcohol consumption had beneficial effects on malnutrition [53] whereas Mathew et al. (2016) found no association between alcohol consumption and malnutrition among older persons in their study [16]. Therefore, further investigations on patterns of alcohol consumption among this population is required to postulate a final conclusion.

Traditionally, Sri Lankan society holds older persons in reverence and provides social and financial support for them [54]. The majority of our participants were married and stayed with their family. In Sri Lanka, provision of older person care is considered to be the responsibility of family members; in large families, care responsibilities might be shared by family members, with each contributing a smaller effort than would be observed in a smaller family [1]. Our results showed that the number of people living with the older person was a protective factor of being at risk of malnutrition. Living with more family members might help older persons to prevent loneliness and social isolation which are common reasons why older persons eat more poorly [55]. Therefore, support from family members may be crucial in nutritional interventions that aim to improve the nutritional status of older persons in the community.

While previous studies have suggested that marital status, level of education, income, tooth loss, use of dentures, chronic illnesses, diabetes, dyslipidaemia, depression, physical activity, eating difficulty, food allergy, loss of appetite and vegetarianism are associated with malnutrition among older persons, our study did not find these factors to be associated with malnutrition [16–19]. A reason for this may be the effect of multiple unidentified confounders which distort or mask actual associations. Although the identified confounders could be controlled, unidentified confounders could not be controlled using statistical analysis [56, 57]. Similarly, a recent systematic review on determinants of malnutrition among community-dwelling older persons highlighted strong evidence that there is no association between various factors and malnutrition. The same review identified poor appetite as the single factor which had a strong association with malnutrition among older persons [14]. Although poor appetite is probably a major cause of malnutrition, it is mediated by a variety of factors such as age, several peptide hormones released by the gut including; ghrelin, CCK, peptide-YY, glucagon-like peptide 1, oxyntomodulin, and pancreatic polypeptide and many neurotransmitters [58–61]. A possible reason for not finding a strong association for loss of appetite with malnutrition in our study might be a result of interactions among the risk factors on appetite among our participants.

### Strengths and limitations

There were a few limitations which warrant consideration in the present study. First, this was a cross-sectional study, which limits the establishment of causality. Second, as the majority of the participants were Sinhalese, our sample may not be representative of the community-dwelling older persons in the country. Third, recall bias may be another limitation. However, the questionnaire was interviewer-administered and probing was used to ensure that the participants recalled information as well as they could. Fourth, non-respondents are likely to be malnourished or at risk of malnutrition [62]. Excluding disabled and mentally-ill older persons may also have affected findings on the prevalence of malnutrition and risk of malnutrition. Fifth, this study did not assess dietary intake which may directly affect the nutritional status of the participants.

On the other hand, to the best of our knowledge, this is the first study on malnutrition conducted among community-dwelling older persons in Sri Lanka. The use of validated and internationally-accepted questionnaires is a strength of the current study. Finally, the use of multistage sampling ensured that a representative sample was recruited from the community.

## Conclusion

Prevalence of risk of malnutrition is high among older persons in the community-dwelling setting. Age, having hypertension and alcohol consumption were significantly associated with malnutrition among the participants. Moreover, the number of people living with the older persons decreased the probability of being at risk of malnutrition. Public health professionals should be encouraged to develop screening strategies according to the identified factors to improve the nutritional status of this vulnerable population.

## Abbreviations

BMI: Body mass index CI Confidence interval MNA-SF Mini nutritional assessment tool – short form OR Odds ratio
